# Entropy Estimation: From Statistical Physics to Classification

**DOI:** 10.3390/ijms27209361

**Published:** 2026-10-21

**Authors:** Federico Vello, Sara Fortuna, Federico Fogolari

**Affiliations:** Dipartimento di Scienze Matematiche, Informatiche e Fisiche (DMIF), University of Udine, Via delle Scienze 206, 33100 Udine, Italy; sara.fortuna@uniud.it

**Keywords:** entropy, kth nearest neighbor method, cross-entropy, classification

## Abstract

Driven by the common entropy formalism in statistical physics and information theory, here we show that methods developed for the analysis of molecular configurational ensembles may be successfully used for classification. Entropy is estimated using the k-th nearest neighbor method combined with the Maximum Information Spanning Tree method to account for the mutual information between variables. This formalism is used to classify new samples using their estimated cross-entropy with the training set. The method is tested using the hand-written digits MNIST and the functional genomics CuMiDa datasets. The results obtained on these diverse datasets are comparable to those obtained by machine learning methods, or better when the number of samples is small. The methods described here have the advantage, compared to other classification methods, of directly linking variables and pairs of variables to the assigned class, a valuable feature when aiming at identifying causal relationships between features and classes (e.g., the relationship between genes and diseases).

## 1. Introduction

Statistical physics aims at linking macroscopic properties of matter with the physics of its microscopic components. In this process fundamental laws of physics are coupled with the laws of probability to derive macroscopic properties through suitable averages over the degrees of freedom of the entities which constitute the system.

Since its inception, with kinetic theory in the mid-eighteenth century, and later with the work of Boltzmann [1] and Gibbs [2], statistical physics made it possible to describe even the most elusive thermodynamic quantity, entropy, in terms of averages over microscopic coordinates. The direct computation of entropy for a molecular system is performed by techniques which use different methods to consider the probability density function (pdf) for continuous variables or the probability mass function (pmf) for discrete or qualitative variables. The continuous and discrete/qualitative variables may require different methods for entropy estimation. Entropy estimation methods may be parametric, based on the expected behavior of molecules, e.g., treated as a collection of independent harmonic oscillators or as non-parametric. The latter may be straightforwardly used for the estimation of entropy for any pdf.

The same formula used by Boltzmann for entropy, save for a multiplicative constant, was independently obtained by Shannon in his mathematical theory of information and, following a suggestion by von Neumann, was also named entropy [3]. The fact that quantities defined in such different contexts and formalisms happen to coincide is far from expected, but also far from being casual. Indeed, the interpretation of entropy as a measure of the average information gained upon knowledge of one symbol of the message is translated immediately into the average information gained upon knowledge of one microstate instead of the macrostate of a system, in classical statistical mechanics. In other words the macrostate plays the role of the unknown symbol, and the microstate plays the role of the specific symbol.

The relationship between thermodynamic entropy and information has been subject of intense debate in the scientific community, starting more than one century ago, with the works of Maxwell and Smoluchowski who imagined devices where information (on the velocity of particles) could be used to violate the second law of thermodynamics [4]. Variations on the theme of Maxwell’s demon have also been proposed in more recent years. The interested reader may refer to the review by Rex [4], which provides a thorough historical account of the subject, and to the works by Landauer [5] and Bennett [6] which consider the physical representation of information. For a different perspective and developments on the subject, the reader may refer to the works of Lairez [7] and Alicki [8].

In information theory Shannon entropy describes the entropy in the same way as in classical statistical physics. Similar information content concepts underpin classification in general, and in particular classification by machine learning (ML), where training, testing, and predictions are performed using the consolidated framework of information theory. Entropy has been used before as an explanatory variable in classification [9,10] and in other contexts to visualize biological sequence information [11], or to classify images [12], just to mention a few diverse applications.

Here, we refer to classification as the task of assigning one out of a number *G* of possible labels to a sample represented by *m* different features, typically real numbers, but also discrete or categorical variables.

Instead of addressing the mapping of statistical learning into statistical physics problems which is a well-established and active area of research (see refs. [13,14,15] and references therein), here we use methods developed and successfully used to compute molecular entropies for classification. Such methods are based on information theory and are completely independent of the physical problems for which they were developed. We consider that the direct linkage between the formalism used in statistical physics to compute entropy and in statistical learning for classificaton provides an important opportunity for cross-fertilization, and we provide an example of this.

We focus on cross-entropy because it is the criterion by which classification is most often performed in ML, and it is natural to wonder whether the tools used for entropy calculation in classical statistical mechanics systems can be borrowed and applied to more general classification problems.

The idea is not unheard of, indeed early methodology developments in the field of ML were driven by analogies with physical systems. In many, but not all, cases the statistical properties of such systems were used.

A physical system is represented by generalized coordinates and by interaction energy parameters. Prototypical of the relationship between physical systems and ML are Hopfield networks [16] where the input and output of a ML task are mapped to the states of the network cells, each of which may assume only two values (±1), and the learning machine is mapped to the set of couplings of a fully connected Ising model (Curie–Weiss model [17]). Such a physical model starting from any input evolves towards the states it was trained on. If part of the input represents the features and another part represents the output, Hopfield networks are able to perform classification.

Statistical physics was very early applied to a similar model (named the “Boltzmann machine”) where the strengths of the connections of the Ising model are modified as to obtain, for a random input, the same Boltzmann distribution as for the training set [18]. In the latter scenario the cross-entropy (or Kullback–Leibler divergence) is a measure of how well the network reproduces the input underlying distribution.

The configurational entropy of molecules may be estimated by a variety of methods [19,20], some of which may be used for different problems under reasonable assumptions concerning the data. Here, we use non-parametric methods for entropy estimation developed in the fields of statistical sciences [21,22,23,24,25,26] and molecular sciences [27,28,29,30,31,32] for classification.

We consider each label of the data as identifying a macrostate and each unlabeled sample as a microstate of a system. Then, for each sample, we estimate the entropy that would result from the sample being assigned to each possible label. Finally, the corresponding minimum entropy label is assigned to the sample. For the above task we use the methods of kth nearest neighbor entropy (kNNe) estimation [21,22,23,24,25,26] and the maximum information spanning tree (MIST) [33,34]. Compared to standard ML classification, the method proposed here has the advantage of being readily interpretable and applicable even when the number of samples is extremely low. The results presented here show that the performance of the method is comparable to more complex approaches like Multilayer Perceptrons or Support Vector Machines.

Among the available entropy estimation methods (parametric and non-parametric) [35,36,37], the kNNe method has distinct advantages [32], balanced off by computational cost, and it is appropriate for continuous variables. Other approaches are more appropriate for discrete variables [38]. The entropy is obviously an ensemble property, but the kNNe method allows for the estimation of the entropy from each sample.

As discussed later, the kNNe method should not be confused with the standard kth nearest neighbor (kNN) method.

In this work a first test is performed on the well-known dataset MNIST (Modified National Institute of Standards and Technology) [39] in order to check for general applicability of the method, for easy rationalization of the results, and for comparison with previous results. MNIST is indeed one of the most used reference datasets for classification.

As a second test we turn to typical Omics research data, e.g., microarray-based gene expression data, which provide a biologically relevant setting for entropy-based classification. In microarray datasets, each sample is characterized by the expression levels of thousands of genes, while the number of available samples is often limited. Moreover, gene-expression variables are not necessarily independent, as coordinated changes in expression may reflect shared regulatory or cellular processes. The joint entropy of these variables describes the collective variability of a multivariate expression profile, accounting for statistical dependencies that are ignored when only marginal entropies are considered. In a supervised classification setting, gene-expression distributions can be characterized separately for each class, and the estimated entropy contribution associated with assigning an unlabeled sample to a candidate class provides a basis for classification. To investigate this approach in a molecular context, we consider the Curated Microarray Database (CuMiDa) [40], which contains gene-expression datasets covering different cancer types and subtypes. The high dimensionality and limited sample sizes that characterize these types of datasets further motivate comparing direct joint-entropy estimation with approximations based on individual features and pairwise mutual information (MI).

## 2. Results and Discussion

### 2.1. Analysis of the MNIST Dataset

The discretization of pixel intensities in the MNIST dataset [39] (described by a probability mass function) prevents the application of the kNNe method because many distances between intensities are 0. For this reason we turn to histogram analysis. It is worth mentioning also that the large number of samples and the low dimensionality (1 and 2) of the spaces considered in the kNNe-MIST analysis make histogramming a much more efficient method to estimate entropy. Therefore, for each of the ten classes (*k*) of the MNIST dataset, the probability distribution function for each pixel (*i*) was estimated as a histogram with constant probability in each bin *j* of width Δx:

(1) $${\hat{p}}_{i}^{k}\left(x\right)=\sum_{j}p_{ij}^{k}h_{j}\left(x\right)$$

where

(2) $$p_{ij}^{k}=\frac{n_{ij}^{k}}{n_{i,tot}^{k}\Delta x}$$

and

$$h_{j}\left(x\right)=\left\{\begin{array}{cc} 1 & x\in \mathrm{bin}\;j\\ 0 & x\notin \mathrm{bin}\;j \end{array}\right.$$

The Kullback–Leibler divergence between the probability distribution of the same pixel *i* in different classes *k* and *l*, $p_{i}^{k}$ and $p_{i}^{l}$ was approximated as follows:

(3) $$D(p_{i}^{k}\left|\right|p_{i}^{l})=\sum_{j}p_{ij}^{k}\log\left(\frac{p_{ij}^{k}}{p_{ij}^{l}}\right)\Delta x$$

**Figure 1:**
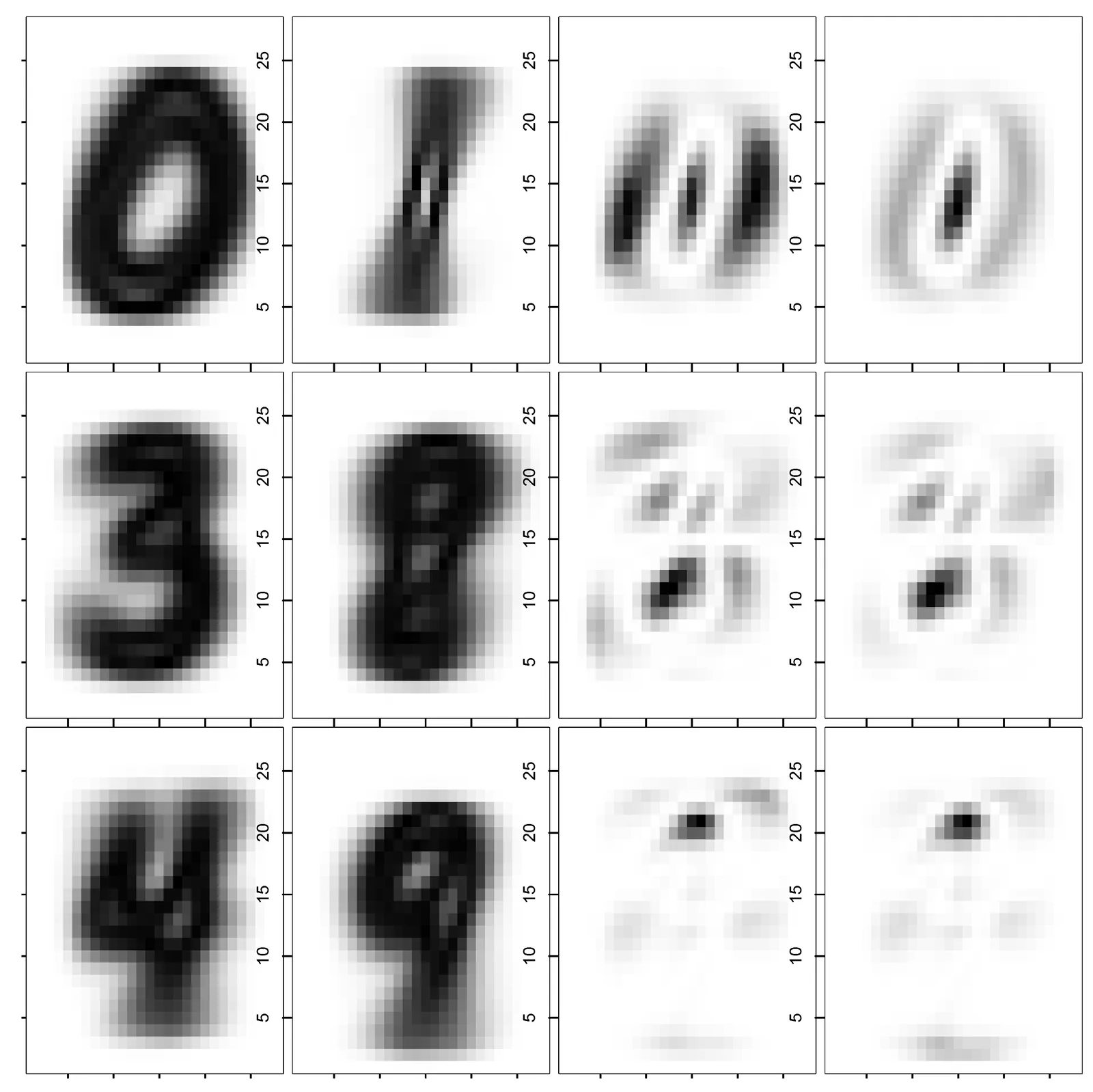
Pixel entropies for digits of a class and Kullback–Leibler divergences between two classes. Starting from the top, rows refer to classes 0 and 1, 3 and 8, and 4 and 9, respectively.

An example of entropy-based analysis of the MNIST training set is reported in Figure 1. Information is relative to the classes 0 and 1, which are very easy to distinguish, and the two most confounded classes 3 and 8 and 4 and 9. The two left-most panels display the entropy of the pixels obtained from the distribution in the training dataset, for the corresponding classes. The right-most panels display the non-symmetric Kullback–Leibler divergences of the pixels in the two distributions.

In the kNNe classification the pixels showing the highest Kullback–Leibler divergence are those who are the most important for classification.

A simple application of the method achieves 0.850 accuracy. The accuracy is comparable to that obtainable with linear modeling. Indeed, classes may be represented as one-hot vectors, i.e., vectors of ten integers which are all zero except the one representing the correct digit which is set to one. With this representation a linear system may be set up and solved in the least-squares sense. The coefficients may be used for predicting the class (i.e., the digit) of test samples. The accuracy achieved by linear modeling is 0.860.

The difference between kNNe and linear modeling is that cross-entropy is explicitly estimated and provides the criterion for classification. Moreover, this is easily extendible, for instance by considering a more refined model for the probability density or probability mass function in this case. Indeed, when only pairs of pixels are considered, MI can be estimated by two-dimensional histograms, and the entropy can be estimated by the upper bound provided by the MIST approach. When this is conducted on the MNIST dataset, an accuracy of 0.934 is achieved. Consideration of pairs of features and their mutual information leads, therefore, to a significant increase in accuracy of 0.084.

In order to check whether the correlations among pixels could be accounted by linear modeling, we also used Singular Value Decomposition (SVD) (see Section 3) to reduce the dimensionality by projecting the data on the first principal components. The accuracy tends to level off at an accuracy of 0.854 at about 100 components. Therefore, reducing the dimensionality does not dramatically improve the accuracy.

**Figure 2:**
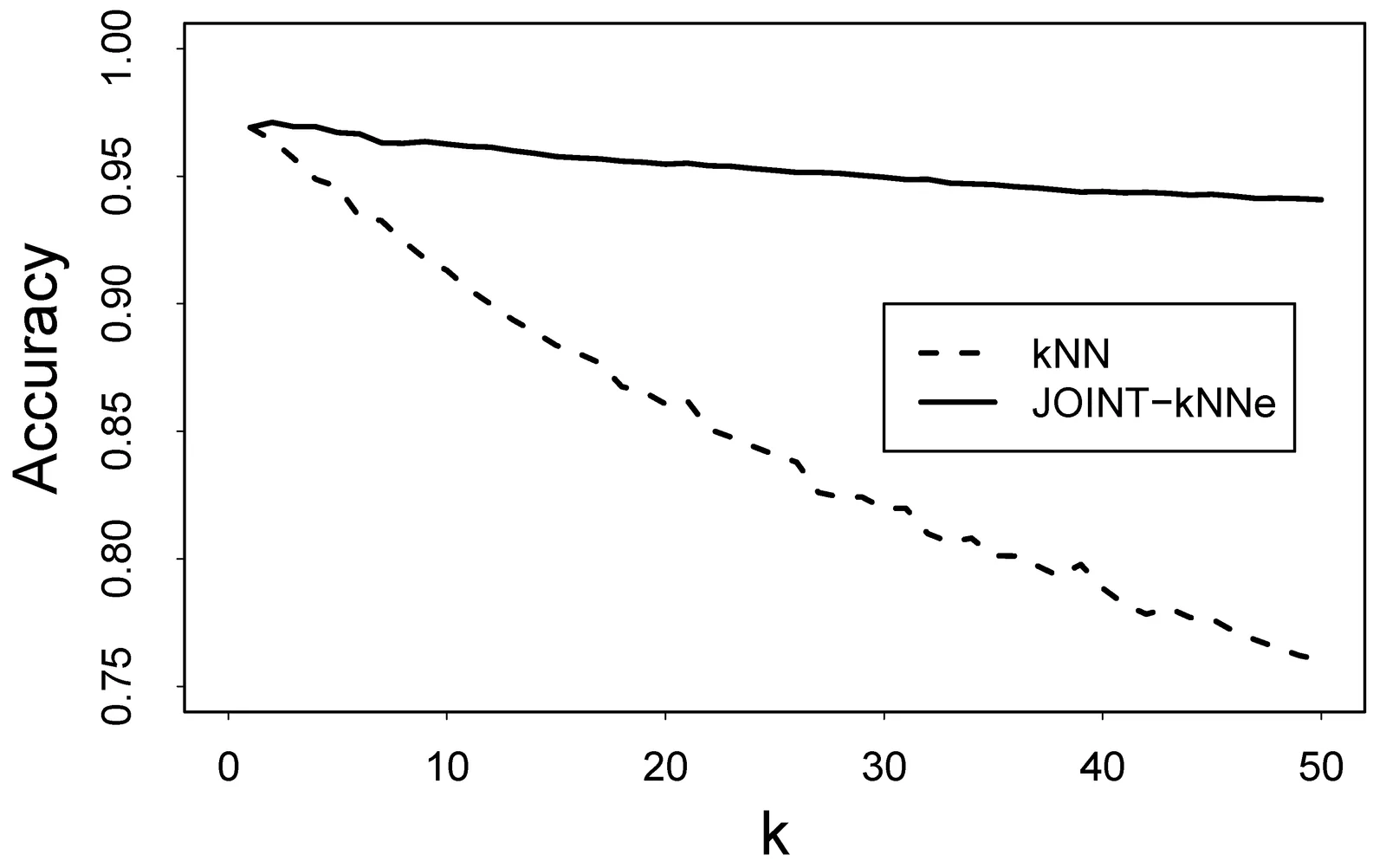
Accuracy of standard kNN and kNNe entropy-based classification.

It is also interesting to compare standard kNN classification with kNNe classification on the MNIST dataset to highlight similarities and differences. For this we consider the accuracy of the classification using increasing values of *k*. For the standard kNN method, the class is assigned based on the most represented class within the first *k* neighbors. For the kNNe entropy-based classification, we estimate the entropy of each sample for each class considering the *k*-th nearest neighbor in the 784-dimensional space of pixel intensities (the procedure is referred to as JOINT-kNNe). So, for the first nearest neighbor, standard kNN and kNNe entropy-based classification produce exactly the same classification. When *k* increases, however, the accuracy of standard kNN classification decreases rapidly (in line with other studies on different datasets [41]) whereas kNNe entropy-based classification slightly increases for the first *k*’s and decreases much slower (Figure 2). It is apparent that, notwithstanding the similarities, the two approaches are different. In the case of standard kNN classification, the first *k* nearest neighbors are taken independently of their class, whereas in kNNe entropy-based classification for each class the first *k* nearest neighbors belonging to that class are considered. For this reason the two approaches essentially coincide for the first nearest neighbor, but they differ for all choices of k≠1.

### 2.2. Analysis of CuMiDa Datasets

#### 2.2.1. Benchmark

CuMiDa datasets provide a good benchmark for the kNNe approach in several respects. First, the datasets contain a small number of samples, sometimes just six samples for each class. In such a scenario, ML methods are prone to overfitting. Second, singling out which genes and which pairs of genes most contribute to classification provides valuable information not available or only indirectly available with ML methods. The genomic analysis of the results is out of the scope of the present work, but we remark that interpretability is a valuable feature of the method proposed here.

To assess the performance of the kNNe classifier, we take advantage of the metrics presented with the data on the CuMiDa website (https://sbcb.inf.ufrgs.br/cumida, (accessed on 10 August 2026)).

The entropy-based classifier achieves accuracies that are broadly comparable to, and in several cases higher than, those reported for standard machine-learning classifiers such as classical kNN, Multi Layer Perceptron (MLP), Random Forests (RFs), and Support Vector Machine (SVM).

Since the datasets contain a different number of samples and classes, we found it useful to sort the datasets by the average number of samples per class, in order to check possible trends in the results.

Only 69 out of the 78 datasets from CuMiDa could be used because some contained only one class or had a samples-per-class ratio less than six.

**Figure 3:**
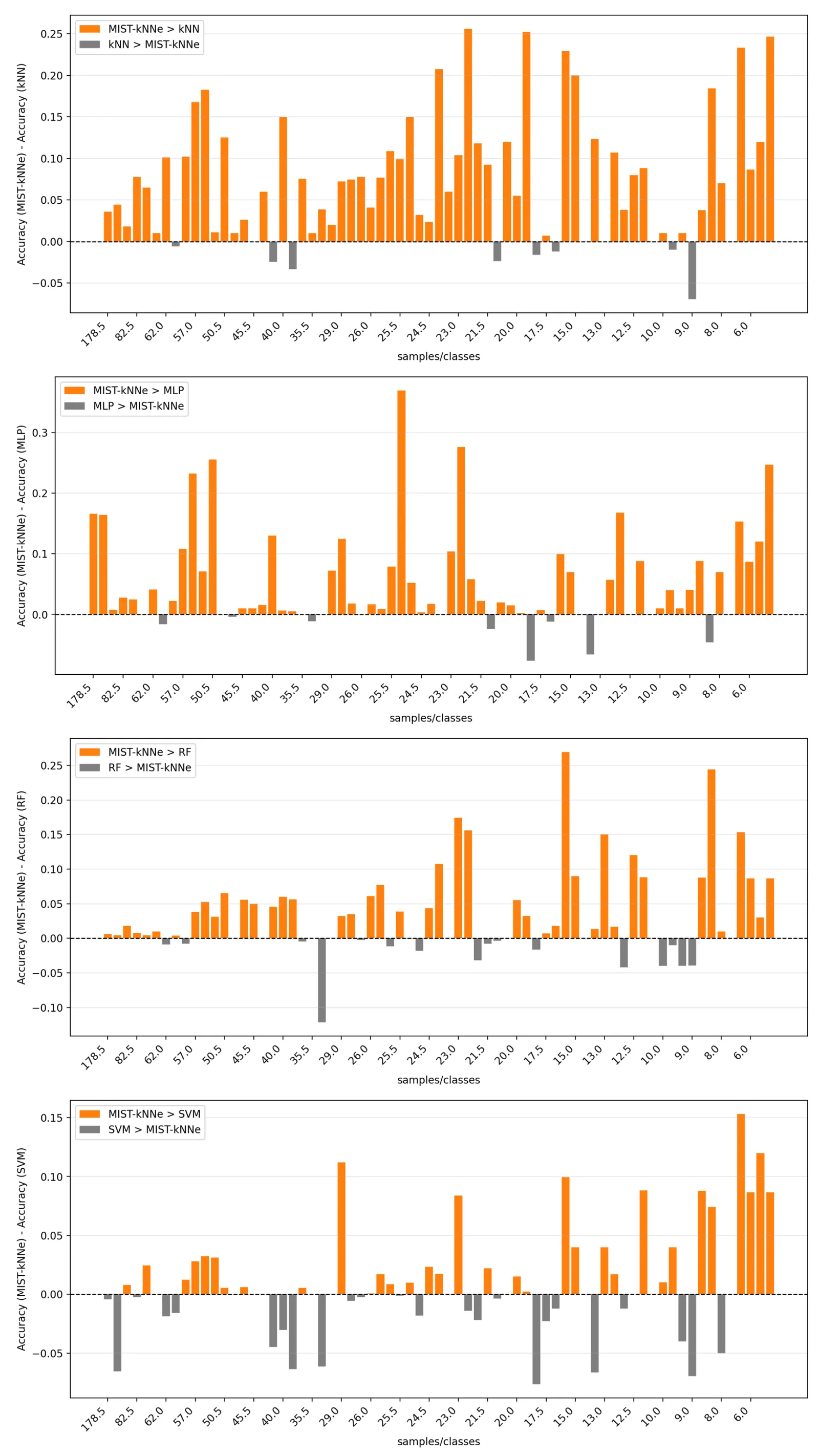
Benchmark of four ML methods presented by CuMiDa. Each graph represents the difference between the MIST-kNNe model and an ML algorithm, including kNN, MLP, RF, and SVM.

Figure 3 compares the four aforementioned methods with our MIST-kNNe model. The comparison was performed using 80 features, since this dimensionality yielded the best average performance after feature reduction based on Analysis of Variance (ANOVA) and Logistic Regression (LR).

The results show that MIST-kNNe achieves higher accuracy than the other methods in most cases, particularly as the number of samples per class decreases. The only consistently competitive method is SVM, which performs slightly better than MIST-kNNe in the leftmost region of the plots, corresponding to datasets with a large number of samples and a limited number of classes.

It should be noted that the accuracy values reported by CuMiDa refer to the application of machine-learning methods without reducing the number of genes. Therefore, the comparison of the reference point with our MIST-kNNe model is also influenced by this methodological difference. As a matter of fact, as discussed in the Feature Dependencies section, dimensionality reduction is an unavoidable step when applying MIST. Indeed, it is particularly evident that when the effective number of training samples per class decreases, namely in the regime where density estimation is intrinsically more difficult and overfitting may more severely affect conventional classifiers, kNNe classification, combined with hybrid-embedded feature selection, is more accurate than standard machine-learning classifiers.

#### 2.2.2. Features Dependencies

We thoroughly investigated the effect of dimensionality reduction across the 69 datasets. Working directly with tens of thousands of features can be problematic for classical machine-learning methods because of the noise introduced by numerous genes. For proper comparison we therefore tested our method using both the original feature sets and reduced subsets of features. The full-features approach was impractical for the MIST algorithm due to the extremely large matrix required for pairwise MI estimation. Consequently, only the SUM-kNNe and JOINT-kNNe analyses were performed using the complete feature sets. By contrast, dimensionality reduction allowed us to apply MIST and evaluate its performance as the number of retained features increased from 20 to 100.

**Figure 4:**
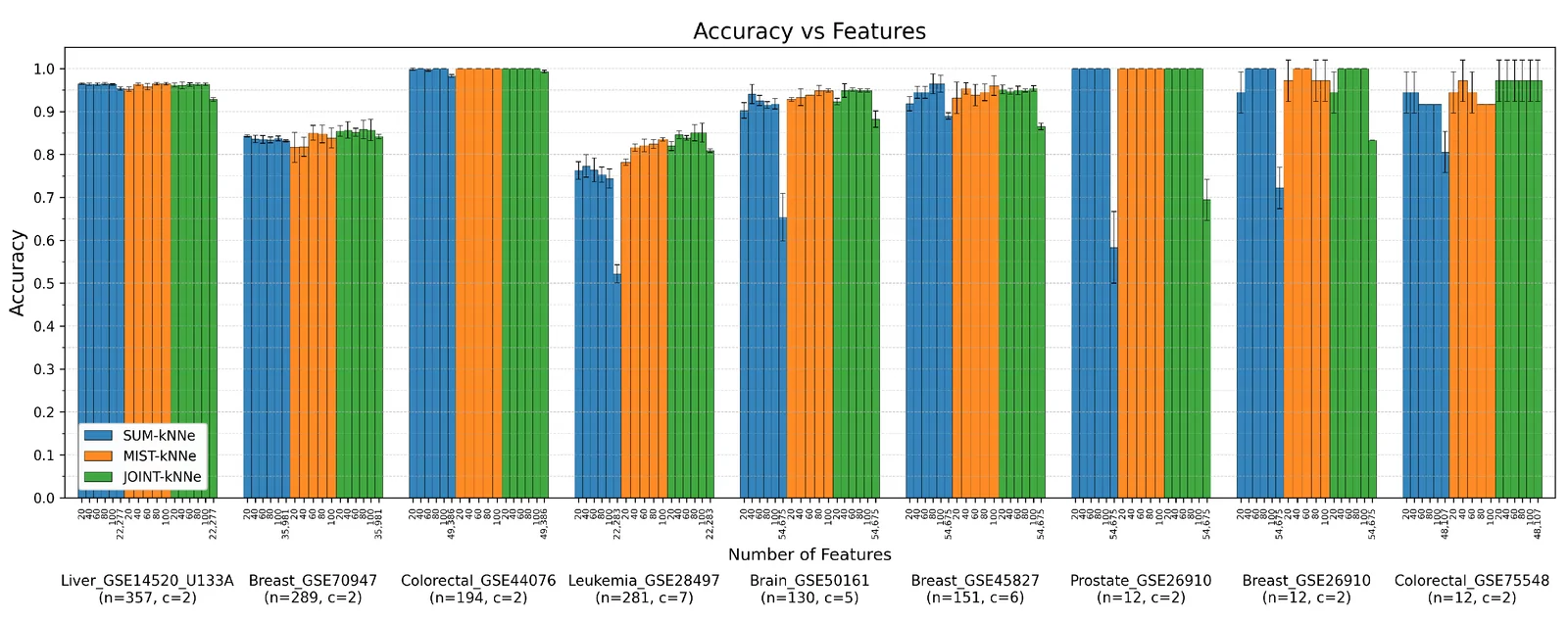
Accuracy vs. number of features obtained via ANOVA + Logistic Regression filtering. SUM-kNNe refers to sum of marginal entropy, MIST-kNNe refers to pipeline including pairwise MI through MIST, and JOINT-kNNe refers to multivariate entropy estimation.

To provide a clear graphical representation of these results (Figure 4), we selected a representative subset of nine datasets according to three criteria: three datasets with a large number of samples and only two classes, three datasets with a large number of samples and multiple classes, and three datasets with a limited number of samples. The complete CSV file containing the results for all 69 datasets is available in the Supplementary Materials.

In multiclass datasets such as Leukemia GSE28497 and Brain GSE50161, MIST-kNNe and JOINT-kNNe systematically improve over the purely marginal SUM-kNNe estimate, reaching accuracies of up to 0.85 and 0.94, respectively. By contrast, on the same datasets, SUM-kNNe achieves lower accuracy and is more sensitive to the number of selected features. The dependence on dimensionality appears to be dataset-specific. In some cases, such as Breast GSE45827, increasing the number of selected features improves the classification accuracy of SUM-kNNe, even beyond that achieved by MIST-kNNe. However, more often, using more than 40 features degrades the performance of SUM-kNNe. This suggests that non-informative or redundant variables can introduce noise unless feature dependencies are explicitly taken into account. The MIST protocol often mitigates this effect by incorporating pairwise mutual-information terms. However, the improvement over SUM-kNNe is not universal, indicating that the relevance of gene–gene dependencies varies substantially across datasets, even under the same feature-selection procedure.

Looking at the last bar of each SUM-kNNe and JOINT-kNNe analysis, representing the complete set of features, it is apparent that the first three datasets can be well classified despite the inclusion of noisy features, which only slightly lowered the accuracy compared to the reduced feature sets. By contrast, when the number of classes increases or the number of samples decreases, the impact of the noise makes the accuracy drop by several percent points, particularly in the SUM-kNNe approach, except for Breast GSE45827.

We analyzed the same group of datasets using Random Forests, instead of ANOVA, pre-filtering followed by Logistic Regression for feature selection. The accuracy achieved was highly comparable to that of the previous approach; however, the computational cost increased substantially, particularly when using 10,000 trees. Consequently, we opted not to pursue this method further.

**Figure 5:**
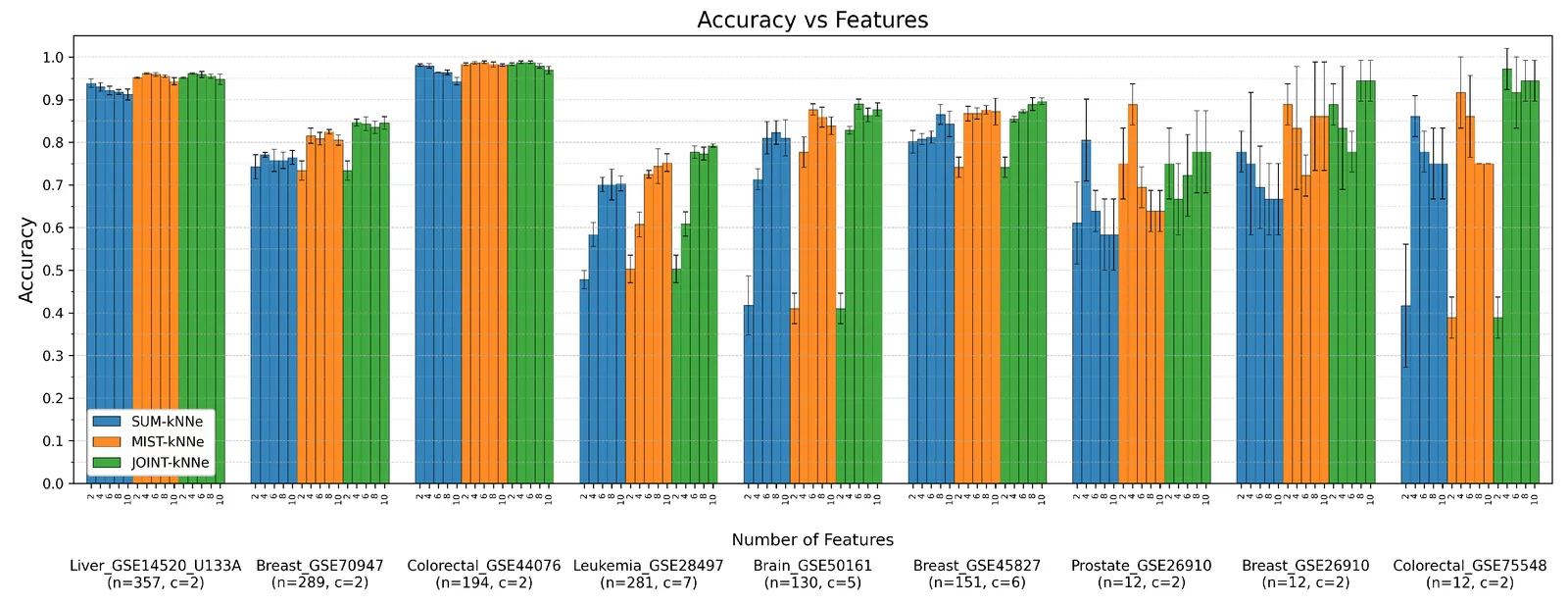
Accuracy vs. number of features (principal components) obtained via SVD. SUM-kNNe refers to sum of marginal entropy, MIST-kNNe refers to pipeline including pairwise MI through MIST, and JOINT-kNNe refers to multivariate entropy estimation.

Despite the initial premise regarding the use of feature extraction (versus feature selection) on tumor gene expression datasets, we nevertheless decided to test its effectiveness. The graph in Figure 5 shows the results of the analyses performed on the same group of datasets also used with feature selection. The first evident aspect is the different range of features used. With SVD, having more than 10 components led to a drastic drop in accuracy, whereas varying the number of components in a range between 2 and 10 yielded results at least comparable to those obtained with ANOVA + Logistic Regression. Comparing Figure 4 and Figure 5, it can be seen that, on average, the accuracy level achieved with feature extraction is lower than that achieved with feature selection. On the datasets with many samples and only two classes, this difference is not too marked, whereas as the number of classes increases and the number of samples decreases, the performance drops significantly, both in terms of accuracy and mean error, in some cases becoming even worse than the full-features approach. It must be said, however, that SVD provides an advantage in terms of computational speed, mainly due to the low complexity of obtaining the $USV^{t}$ matrix factorization and the use of a number of features an order of magnitude lower than that required by feature selection.

JOINT-kNNe is frequently the best-performing protocol. This result was not obvious *a priori*, since JOINT-kNNe could have been expected to suffer more severely from the curse of dimensionality, with distances increasing relevantly as the number of features grows. Nevertheless, this gain comes at the cost of reduced interpretability. In contrast, MIST-kNNe provides a sparse representation of the dominant mutual-information links between genes, an aspect that is particularly relevant in oncological applications, where both predictive accuracy and biological interpretability must be considered.

## 3. Materials and Methods

### 3.1. Entropy Estimation

We assume samples *x* of a multivariate variable *X* are randomly drawn by *M* sources (classes) with probability density functions $\rho_{m}\left(x\right)$ and probabilities $p_{m}$, respectively. We refer to the general distribution as ρ(x,m).

The entropy of the distribution is as follows [42]:

(4)S=−∑m=1,M∫ρ(x,m)log(ρ(x,m))(5)  =−∑m=1,M∫pmρm(x)log(pmρm(x))(6)  =−∑m=1,M∫pmρm(x)log(pm)−∑m=1,M∫pmρm(x)log(ρm(x))(7)  =−∑m=1,Mpmlog(pm)−∑m=1,Mpm∫ρm(x)log(ρm(x))(8)  =−∑m=1,M<log(pm)>−∑m=1,Mpm<log(ρm(x))>(9)  =Sgroups+∑m=1,MpmSm

where $S_{groups}$ is the entropy due to class distribution, and $S_{m}$ is the entropy of the distribution of samples in class *m*.

We consider now the estimation of entropy from *n* samples. This is expressed as an average over the samples. The entropy term $S_{groups}$ is estimated by the standard formula using Miller–Madow correction [43]:

(10) $${\hat{S}}_{groups}=-\sum_{m=1,M}\frac{n_{m}}{n}\log\left(\frac{n_{m}}{n}\right)+\frac{M-1}{2n}$$

The entropy terms $S_{m}$ are estimated using the *k*-th nearest neighbor method [21,22,32]:

(11)S^m,k=∑i=1,nmlog(Vi,km)+log(nm)−Lk−1+γnm(12)=∑i=1,nlog(Vi,km)nm+log(nm)−Lk−1+γ

where $V_{i,k}^{m}$ is the volume of a ball with radius equal to the distance of the k-th nearest neighbor of class *m* from the *i*-th sample in class *m*.

Classification amounts to assigning (deterministically or not) to a new sample *x* the class *l*, which may be different from its true class, say *t*.

The rule by which classification, based on training examples, is performed, establishes a distribution η(x,m) which will be in general different from the true (unknown) distribution ρ(x,m).

The sum of Equations (10) and (12) provides, as shown above, an unbiased estimator of the quantity $<-\log\left(\eta \left(x,m\right)\right){>}_{\rho }$, which is in general different from the entropy of the true and estimated distribution ρ(x,m) and $\hat{\rho }\left(x,m\right)$, but also from the entropy of the distribution η(x,m) because samples are drawn from the true distribution ρ(x,m), as indicated by the subscript for the average brackets. The distributions ρ(x,m) and η(x,m) are actually the same when the classifier assigns to each sample *x* the class *m* with the same probability.

The expected change in the estimated entropy, with respect to the unknown true entropy, estimated by Equations (10) and (12) is given by the following equation:

(13)ΔS^=−∑m=1,M∫ρ(x,m)log(η(x,m))−∫ρ(x,m)log(ρ(x,m))(14)=∑m=1,M∫ρ(x,m)log(ρ(x,m)η(x,m))

The last line is the Kullback–Leibler distance $D_{KL}\left(\rho \left(x,m\right)||\eta \left(x,m\right)\right)$ between the probability distributions ρ(x,m) and η(x,m), which is greater than zero by the Information Theorem, i.e.,

(15) $$\Delta \hat{S}\geq 0$$

with the equal sign holding if and only if ρ(x,m)=η(x,m) [42].

The latter inequality provides a criterion to assign the class to a new sample, by choosing the class providing the smaller positive change in the estimated entropy.

The reference $S_{true}$ is estimated using Equations (10) and (12). Since $S_{true}$ is equal for all possible class assignments, we just need to choose the assignment *l* which minimizes the entropy estimate associated with the new sample, i.e.,

(16) $$k=\arg\min_{l}\left(-\log\left(\frac{n_{l}}{n}\right)+\left(\log\left(V_{n_{l},k}\right)+\log\left(n_{l}\right)-L_{k-1}+\gamma \right)\right)$$

For the sake of completeness, it should be noted that assigning a new sample to class *l* would also result in a change of the estimate of the entropy based on the sole training set. Using Equation (16) is, however, extremely convenient because neighbors of the train samples are not recomputed. No sizable difference was observed in our tests using Equation (16) compared to recalculating the nearest neighbors for the training set.

Throughout this paper we will refer to kNN entropy-based classification as kNNe classification.

For multidimensional spaces kNNe may be applied under the approximation that all variables are independent, and, therefore, the entropy is just the sum of each variable pdf entropy, which is referred to as SUM-kNNe. Alternatively, we refer to JOINT-kNNe when considering the euclidean distances in the multidimensional space.

### 3.2. Maximum Information Spanning Tree

In high dimensional spaces the kNNe method, as described above, faces the problem that the distances on which the method is based on result from the combination of informative and much less informative variables. For this reason the straightforward application of the method, which we named JOINT-kNNe, may be inappropriate. For this reason other approximate ways to estimate the entropy of multivariate pdfs have been proposed. In the field of molecular configurational ensembles, two main approaches based on mutual information have been used: (i) a mutual information expansion (MIE), truncated at pairwise mutual informations, which has demonstrated its potential for torsional variables [28,44,45], but suffers from quadratic growth of terms, many of which are just adding noise; and (ii) the maximum information spanning tree (MIST) method, proposed by Tidor and collaborators [33,34], which also accounts for mutual information between variables. The MIST approach considers pairwise mutual information and constructs a tree corresponding to a chain of conditional entropies, yielding the lowest upper bound on the entropy that can be obtained from mutual information involving at most two variables. In the approach for an *n*-dimensional space, only n−1 mutual informations are taken into consideration. This approach has also been extended to group variables and to evaluate the mutual information between pairs of groups [46]. The entropy-based classification method using the kNN method to estimate univariate entropies and pairwise mutual information is referred to as MIST-kNNe.

### 3.3. Choice of the Neighborhood

The choice of *k* is crucial as it effectively represents the main tunable parameter in the *k*-th nearest neighbor entropy estimation. It is well known that choosing a small value of *k* provides stronger support for the assumption that the probability density is approximately uniform within the ball. On the other hand, this choice leads to greater variance of the entropy estimate [32]. An alternative is to choose a larger value of *k*. In this case, the entropy estimates obtained for different points of the distribution tend to become more uniform due to the reduced variance. However, this introduces a bias, since the distribution within a ball of larger radius may not be homogeneous, thereby reducing the validity of the underlying assumption. Moreover, smoothing a non-uniform density may result in larger entropy estimates. To mitigate this issue, we adopted an intermediate approach: entropy was estimated for several values of *k* (from 1 to 5 by default), and the median of the resulting entropy estimates was then used in order to reduce the dependence on any single neighborhood size and limit the influence of unusually high or low estimates. For the CuMiDa datasets, this strategy also provides a common procedure that can be applied consistently across datasets with markedly different sample sizes. When necessary, for example for datasets including only 12 samples, the admissible range of *k* is automatically restricted according to the number of samples in the least-populated class of the corresponding training set, so that only valid neighborhood sizes are considered ($k\leq n_{\mathrm{class}}-1$). Thus, the use of k=1,…,5 is not intended to identify the accuracy-maximizing value of *k* for each individual dataset, but rather to provide a robust compromise between the variance associated with very small neighborhoods and the bias introduced by larger ones. For a large dataset such as MNIST, a nested cross-validation procedure for tuning *k* would in principle be feasible. However, in that case our purpose was not to optimize a single value of *k*, but rather to explore the behavior of the classifier over different neighborhood sizes and to assess the dependence of the results on *k*.

### 3.4. Datasets

#### 3.4.1. Randomized MNIST

Since its original assembly by LeCun et al. [39], the MNIST dataset has become a standard benchmark for testing ML classification algorithms. The dataset consists of 60,000 28 × 28 pixel images representing hand-written digits, converted to arrays of 784 integers in the range 0 to 255. If the indices of the pixels are randomized, the correlations among the pixels cannot be derived by their indices anymore. Algorithms specific for images, like rotations, distortions, convolutions, etc., cannot be applied in a straightforward way on the randomized MNIST dataset, and we are left with the more general problem of classifying a set of 784-integer arrays into one of ten classes. The choice of randomizing the array components’ indices adopted in the present work stems from the requirement to test a general and not image-specific classification method, thus not using knowledge that the arrays represent images. On the other hand, reordering the indices allows for easy visualization of the contributions of single pixel to classification of digits or their disambiguation.

The set is characterized by a limited number of features, i.e., the 784 pixels, and by a large number of training examples (60,000) and a moderate set of classes (10).

Also, it is worth noting that there are always many pixels contributing information that is able to discriminate among different classes, i.e., whose probability distributions differ significantly between different classes.

For the same set a large number of classifiers has been tested, and results are reported in the original paper by LeCun et al. [39].

#### 3.4.2. CuMiDa

The Curated Microarray Database (CuMiDa) [40] is a database of 78 hand-curated microarray data sets selected from more than 30,000 microarray experiments from the Gene Expression Omnibus [47]. The sets were selected for different cancer subtypes of colorectal, gastric, pancreatic, liver, bladder, lung, throat, renal, brain, prostate, ovary, leukemia, and breast cancers. The experiments were chosen in such a way that no interference (e.g., by drugs, microRNA, small interfering RNA, engineered mutations, etc.) with the expression levels of the cells was performed during the experiments and that at least six samples were present in the set.

The work describing CuMiDa reports further details and classification accuracies obtained by different classifiers, which are used in the present work for comparison with the methods presented in this work [40].

### 3.5. System Dimensionality Reduction

General classification tasks pose a significant challenge because datasets may contain a small number of samples versus a massive number of features. Many of the features may be irrelevant or redundant, and their consideration into algorithms may result in overfitting or noise addition. For these reasons, besides the ensuing reduction in computational cost, the reduction in system dimensionality is typically performed as a pre-processing step in classification pipelines.

The ways to achieve this tasks are the subject of a wide research area. Here, we use feature extraction by projecting over the first principal components and feature selection ranging from the simple between-class variance method to more accurate Random Forests (RFs), followed by Logistic Regression, in order to obtain hybrid-embedded filter methods. The latter methods are also used as classification methods, but here they are used only for feature selection.

The implemented pipeline within the main execution script evaluates these classification algorithms using a Stratified K-Fold cross-validation as implemented in the python software Scikit-learn v. 1.7.2 [48]. To prevent data leakage, feature selection is performed using exclusively the training data during each fold before proceeding to classification of the test set cases.

#### 3.5.1. Feature Reduction

##### SVD

Singular Value Decomposition [49] was used as a feature extraction method to project the original data onto a lower-dimensional space. Given a centered data matrix $X\in R^{n\times p}$, containing *n* samples and *p* features, its reduced SVD can be written as

(17) $$X=U\Sigma V^{T},$$

where $U\in R^{n\times r}$ and $V\in R^{p\times r}$ have orthonormal columns, r=rank(X), and $\Sigma =\mathrm{diag}(\sigma_{1},\ldots ,\sigma_{r})$ contains the singular values ordered as $\sigma_{1}\geq \sigma_{2}\geq \cdots \geq \sigma_{r}\geq 0$.

For centered data, the connection with Principal Component Analysis (PCA) follows from

(18) $$C=\frac{1}{n-1}X^{T}X=V\frac{\Sigma^{2}}{n-1}V^{T},$$

so that the columns of *V* are the principal directions and the variance associated with component *i* is $\lambda_{i}=\sigma_{i}^{2}/\left(n-1\right)$. The fraction of the total variance accounted for by the first $N_{\mathrm{comp}}$ components is therefore

(19) $$R\left(N_{\mathrm{comp}}\right)=\frac{\sum_{i=1}^{N_{\mathrm{comp}}}\sigma_{i}^{2}}{\sum_{i=1}^{r}\sigma_{i}^{2}}.$$

Dimensionality reduction was performed by retaining the first $N_{\mathrm{comp}}$ right singular vectors and projecting the samples as

(20) $$X_{\mathrm{red}}=XV_{N_{\mathrm{comp}}}=U_{N_{\mathrm{comp}}}\Sigma_{N_{\mathrm{comp}}}.$$

Within each cross-validation fold, centering and SVD were determined exclusively from the training data, and the corresponding transformation was subsequently applied to the test data, thus preventing data leakage.

Although SVD efficiently reduces the dimensionality of high-dimensional data, each resulting component is a linear combination of the original features and therefore does not retain a direct correspondence with individual variables. For this reason, here feature selection was generally preferred for the CuMiDa datasets. Retaining the original variables allows the information relevant for classification to be associated directly with individual genes and, in the MIST analysis, with specific pairs of genes, thus preserving the biological interpretability of marginal and pairwise contributions.

#### 3.5.2. Feature Selection

##### ANOVA

Analysis of variance (ANOVA) [50] was used as a univariate filter to rank the original features according to their ability to discriminate among the classes. For each feature *j*, the mean value $\mu_{c,j}$ within each class *c* was compared with the global mean $\mu_{j}$. The between-class and within-class sums of squares were computed as

$$SS_{j}^{\mathrm{between}}=\sum_{c\in C}n_{c}{\left(\mu_{c,j}-\mu_{j}\right)}^{2},\;SS_{j}^{\mathrm{within}}=\sum_{c\in C}\sum_{i:y_{i}=c}{\left(x_{i,j}-\mu_{c,j}\right)}^{2},$$

where $n_{c}$ is the number of samples in class *c*. The final score assigned to feature *j* was the standard one-way ANOVA *F*-score,

$$F_{j}=\frac{SS_{j}^{\mathrm{between}}/\left(|C|-1\right)}{SS_{j}^{\mathrm{within}}/\left(n-|C|\right)}.$$

Features with larger $F_{j}$ values were considered more informative, since they display large separation between class means compared to the dispersion within each class. The features were ranked by decreasing $F_{j}$, and the first $N_{\mathrm{feat}}$ features were retained for the subsequent entropy-based classification step. The ranking was computed independently within each training fold, before retaining the same selected feature subset to the corresponding test fold.

##### Random Forests

For Random Forests (RFs) [51] the columns in the training dataset are randomly resampled to provide a large number (we tested 1000 or 10,000) of different datasets on each of which a decision tree is built. The split at each decision tree is performed by randomly taking into account only a subset of features (we used the square root of the total number of samples). The criterion employed to choose the splitting feature and the splitting threshold is the class entropy of the feature, i.e., for feature *j* it is as follows:

$$h_{j}=-\sum_{c=1}^{\left|C\right|}{\hat{p}}_{c,j}\log\left({\hat{p}}_{c,j}\right)$$

where ${\hat{p}}_{c,j}=\frac{n_{c,j}}{\sum_{c=1}^{\left|C\right|}n_{c,j}}$

For each chosen feature the optimal threshold for splitting is found, and the feature and threshold leading to the largest reduction (Δh) in entropy between the parent node and the nodes resulting from splitting is chosen, i.e.,

$$\mathrm{chosen}\;j=\underset{k}{\mathrm{argmax}}\left(h_{k,\mathrm{parent}\;\mathrm{node}}-w_{\mathrm{left}}h_{k,\mathrm{left}\;\mathrm{node}}-w_{\mathrm{right}}h_{k,\mathrm{right}\;\mathrm{node}}\right)$$

Note that at the descending nodes, the entropy is weighted by the fraction of samples at the node.

Features are finally ranked by importance by summing the normalized decrease in entropy at all nodes using each feature. The decrease in entropy represents the information gain at each node. The importance of feature *j* is computed as follows:

$$I_{j}=\frac{\sum_{\mathrm{trees}}\sum_{\mathrm{nodes}\;\mathrm{using}\;j}\Delta h}{\sum_{\mathrm{trees}}\sum_{\mathrm{all}\;\mathrm{nodes}}\Delta h}$$

The features are selected up to a chosen importance threshold in order to retain a fixed number of features.

##### Random Forests/ANOVA + Logistic Regression

To power up the feature selection, two hybrid-embedded methods have been included. The initial set of features has been pre-filtered using Random Forests or ANOVA, in order to retain 0.01 of the features. The latter features were further filtered using Logistic Regression. Logistic Regression [52] is applied using an elastic net regularized with mixing coefficient 0.5. The importance of feature *j* is finally computed as follows:

$$I_{j}=\left|a_{j}\right|$$

where $a_{j}$ is the coefficient of the Logistic Regression, in the case when there are only two classes or as follows:

$$I_{j}=\max_{c}\left|a_{j}^{c}\right|$$

where *c* runs over the classes, and $a_{j}^{c}$ is the Logistic Regression coefficient of feature *j* for the binary Logistic Regression, where class *c* is singled out from the other ones.

##### Accuracy

In this work we report the accuracy of classification as the ratio of the number of samples for which the class is correctly guessed over the total number of samples. This simple definition has the advantage of being intuitive, and it allows for comparison with most previous studies. It should be mentioned that the accuracy defined in this way is by no means the only possible nor perhaps the most appropriate quantity to assess the performance of a classifier. For an example of different definitions of accuracy, see ref. [38].

## 4. Conclusions

The k-th nearest neighbor method for entropy estimation (kNNe), developed in the context of entropy estimation for molecular configurational ensembles, has been applied to the task of classification. The method enables the estimation of the entropy from each sample. The estimated cross-entropy with the pdf of train samples of each class is used to classify test samples.

The results obtained on diverse datasets, with largely different number of samples and classes, show that the method is comparable with ML methods, especially when the number of samples is small, a situation where methods based on a large number of tunable parameters are expected to be prone to overfitting.

For practical application of the method, in particular the application of MIST, which requires the mutual informations of all pairs of features, it is necessary to reduce the dimensionality of the samples. In the cases considered here, a simple ANOVA analysis gave results similar to those obtained using a computationally more intense method like Random Forests. Feature selection and the choice of MIST for taking into account mutual information, versus other entropy approximation methods, was motivated by the need of highlighting the contribution of relevant features over noisy irrelevant features.

The main advantage of the kNNe method with respect to other classification methods resides in the straightforward theoretically grounded linkage of features with the assigned class, which makes the method useful not only for classifying samples but also to identify most relevant features (and pairs of features) of the sample. The method is therefore fully interpretable, making it a useful alternative or a complementary tool to existing ones, for classification.

## Data Availability

The data analyzed in this study are available in Hugging Face Datasets at the following https://huggingface.co/datasets/ylecun/mnist, (accessed on 10 August 2026) and in CuMiDa Database at the following https://sbcb.inf.ufrgs.br/cumida, (accessed on 10 August 2026).

## Supplementary Materials

The following supporting information can be downloaded at https://www.mdpi.com/article/10.3390/ijms27209361/s1, and is also available at https://github.com/federicovello18-droid/Entropy-Classification/blob/main/supplementary_files.zip, (accessed on 25 August 2026). The CSV files contain the classification results obtained across all 69 analyzed CuMiDa datasets. One file contains the results obtained using ANOVA + Logistic Regression for feature selection, alongside the full-feature analysis, while the other contains the results following feature extraction via SVD. Each row corresponds to a specific number of features and reports all the parameters used for the analysis, including the actual number of features and the effective range of k. In addition to the average accuracy computed over three repetitions, Cohen’s Kappa is also provided as an evaluation metric (which is not reported in the main manuscript).

## Abbreviations

The following abbreviations are used in this manuscript:

pdf	probability density function
kNNe	k-th nearest neighbor entropy estimation
MIST	maximum information spanning tree
SUM-kNNe	k-th nearest neighbor entropy estimation using the sum of single variable entropies.
MI	mutual information
MIST-kNNe	k-th nearest neighbor entropy estimation using the sum of single variable entropies and the mutual information corresponding to edges in the MIST.
JOINT kNNe	k-th nearest neighbor entropy estimation applied in the multidimensional space of all variables
MIE	mutual information expansion
MNIST	modified national institute of standards and technology
CuMiDa	curated microarray database
SVD	singular value decomposition
PCA	principal component analysis
ANOVA	analysis of variance
kNN	k nearest neighbors method for classification
SVM	support vector machine
RF	random forest
MLP	multilayer perceptron
ML	machine learning
